# Microbiota of the Oropharynx and Endoscope Compared to the Esophagus

**DOI:** 10.1038/s41598-019-46747-y

**Published:** 2019-07-15

**Authors:** Ikenna C. Okereke, Aaron L. Miller, Catherine F. Hamilton, Adam L. Booth, Gabriel L. Reep, Clark L. Andersen, Sandy T. Reynolds, Richard B. Pyles

**Affiliations:** 1 Division of Cardiothoracic Surgery, University of Texas Medical Branch, Galveston, TX USA; 2 Department of Microbiology and Immunology, University of Texas Medical Branch, Galveston, TX USA; 3 Division of Pathology, University of Texas Medical Branch, Galveston, TX USA; 4 Division of Gastroenterology, University of Texas Medical Branch, Galveston, TX USA; 5 Department of Biostatistics, University of Texas Medical Branch, Galveston, TX USA

**Keywords:** Barrett oesophagus, Bacteriology, Clinical microbiology

## Abstract

The role of the microflora in the development of esophageal disease is still largely unknown and is being investigated in more detail. Our goal was to determine how the microbiota levels of endoscope and uvular swabs compared to the levels of tissue biopsies along various points of the esophagus. 17 patients with Barrett’s esophagus agreed to participate in the study. Biopsies of esophageal mucosa were taken from the (1) proximal esophagus, (2) mid-esophagus, (3) distal esophagus, and (4) Barrett’s esophagus. Swabs were also taken from the uvula and the endoscope. Throughout the esophagus, 17 bacterial genera were detected from the samples. The microflora pattern obtained from the uvula and endoscopic swabs did not correlate well with mucosal biopsies along any aspect of the esophagus. There were statistically significant differences in the levels and proportions of bacteria found when comparing the uvula swab to the esophageal biopsies and when comparing the endoscope swab to the esophageal biopsies. Obtaining a simple swab of the uvula or endoscope itself appears to be a poor substitute for tissue biopsy of esophageal mucosa when evaluating microflora patterns. When performing microflora studies of the esophagus, mucosal biopsies should be used for analysis.

## Introduction

The role of intraluminal microbiota in causing disease in the gastrointestinal tract is increasingly being studied^1,2^. Various diseases in other aspects of the gastrointestinal tract have well-known associations with specific microbiota, such as *Helicobacter pylori* with peptic ulcer disease and gastric mucosal-associated lymphoid tissue (MALT) lymphoma^3–5^. Diseases such as eosinophilic esophagitis and esophageal adenocarcinoma also have been associated with particular microbiome expression patterns^6,7^. There are several methods which can be used to collect specimen, and some centers have used a sample from one aspect of the gastrointestinal tract as a wide surrogate of representation of the microbiome of the entire gastrointestinal tract^8–10^. But it has been shown that the microbial composition varies at different regions of the esophagus, likely as a result of the differences in the intraluminal environment in the proximal versus distal esophagus^11,12^.

There is still debate about the most reliable technique to analyze the microbial composition in the esophagus. Endoscopy with mucosal biopsy is the most common technique used, but this procedure requires anesthesia for the patient, is invasive and has a small but existent risk of causing bleeding within the esophagus. Performing the analysis using a swab of the endoscope or the oropharynx would be less invasive, but previous literature has not investigated how similar these swabs are compared to analysis on mucosal tissue. Our goal was to determine how similar the microbial composition was on oropharyngeal and endoscope swabs compared to mucosal biopsies.

## Materials and Methods

### Endoscopy

After obtaining institutional review board approval (University of Texas Medical Branch Review board, IRB # 17-0215), 17 patients with Barrett’s esophagus agreed to participate and were included in the study. Three of the patients had low-grade dysplasia, while the remaining patients had no dysplasia. The study was performed in accordance with institution regulations. Patients with Barrett’s esophagus were chosen because they were scheduled to receive routine surveillance endoscopy for Barrett’s esophagus as clinically indicated. All patients with a known diagnosis of Barrett’s esophagus who were scheduled to undergo surveillance biopsy and agreed to participate in the study were included. Informed consent was obtained from each patient.

Prior to its use, the endoscope was sterilized and placed in a sterile container. The endoscope was then immediately placed into the esophagus without contacting any other surfaces, and swabbed immediately following removal from the esophagus. During the endoscopy, biopsies of the esophagus were taken from the (1) proximal esophagus, (2) mid-esophagus, (3) distal esophagus, and (4) Barrett’s esophagus and collected for research purposes. Swabs were then taken of the uvula and of the endoscope itself, using a sterile swab. The biopsies of the esophagus and the swabs were performed prior to the esophagus ever entering the stomach. The endoscope was then re-inserted and the remaining portion of the surveillance endoscopy was completed.

### Tissue swabs

A tissue swab was taken from its sterile packaging and applied to (1) the uvula and (2) the endoscope. The swab of the uvula was obtained at the uvula only, taking precautions to avoid contact with the tongue or the palate. The swab of the endoscope was taken after the endoscope was passed through the oropharynx to the distal esophagus but before tissue biopsies were performed and before the endoscope entered the stomach, to prevent the endoscope from being exposed to the acidic environment of the stomach.

### RNA extraction

The samples and swabs were immediately placed into a sterile lysing tube, treated with a lysis buffer and homogenized using a bead mill homogenizer. RNA extraction was performed using RNeasy Mini Kits (Qiagen, Hilden, Germany). The concentration and purity of the RNA was assessed using ultraviolet spectroscopy and gel electrophoresis.

### Microflora array

**Figure 1:**
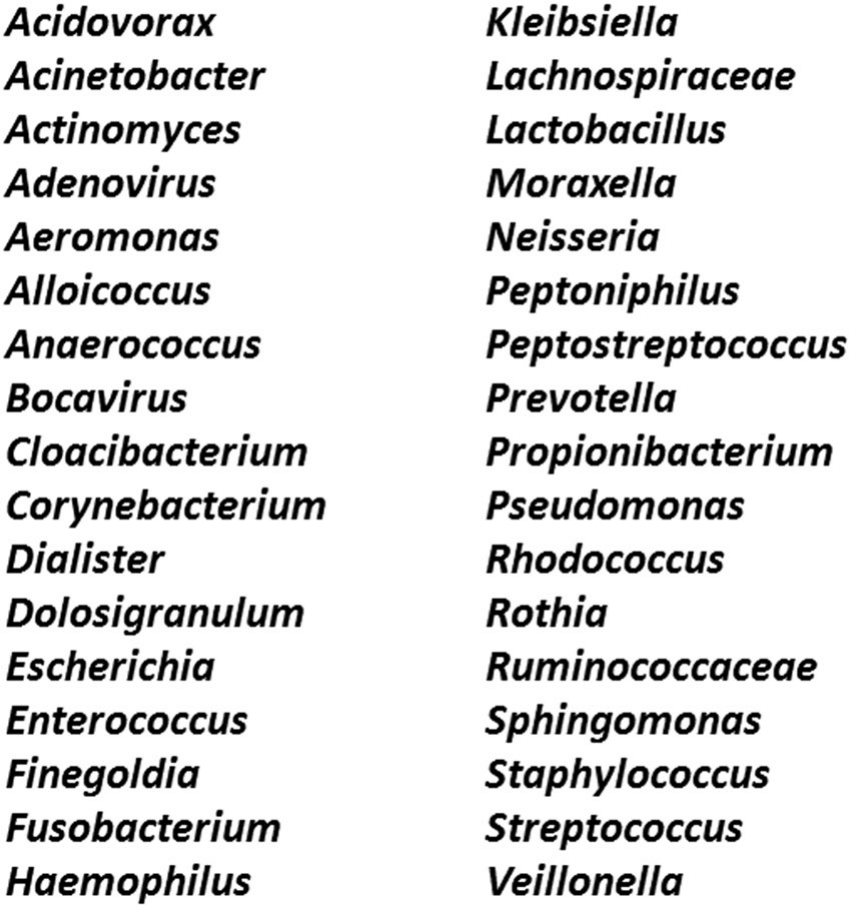
List of bacterial genera in Nasal Microbiome Array.

All biopsies and swabs were evaluated using a quantitative polymerase chain reaction (qPCR) panel (Nasal Microbiome Array, Fig. 1) targeting 34 individual bacterial genera from the upper respiratory tract originally identified using next generation 16 s meta-genomic sequencing. All qPCR targets were confirmed via conventional Sanger sequencing and melt temperature matched to historic controls. Universal 16 s was used as a load for gross bacteria level and hGAPDH was used to determine overall sample integrity.

### Sequencing

Sample sequencing was carried out using a fusion-PCR method. Briefly, fusion-primers were designed in accordance with the manufacturer’s guidelines (Ion Amplification Library Preparation – Fusion Method, Life Technologies, Carlsbad, CA) using Ion Xpress Barcodes linked to 16 s gene primer pairs targeting hyper-variable regions 1–8^13^. Each 25 µl PCR was carried out using: 12.5 µl iQ supermix^™^ (Bio-Rad, Hercules, CA), 1 µl of both forward and reverse (5 µM) primers, 9.5 µl nuclease-free water and 1 µl of DNA template. DNA from each patient from each sample (uvula swab, endoscope swab, proximal esophagus, mid-esophagus, distal esophagus, Barrett’s esophagus) were used as a template for creation of subsequent fusion 16 s libraries. PCR was completed in a c1000 thermocycler (Bio-Rad) using the following parameters: Cycle 1), 95 C, 3 minutes, Cycle 2), Step 1–95 C, 45 seconds; Step 2—Primer-specific annealing temps., 45 seconds; Step 3–72 C 2:00, repeat 39x; Step 4–72 C for 7:00. PCR products were purified using Qiagen Qiaquick spin-columns and quantified using a spectrophotometer (Bio-Rad). PCR products were then diluted, mixed in equal proportion and sequenced on an Ion Torrent GeneStudio S5 System using Ion 520 sequencing kits together with 520 size chips following the manufacturer’s instructions (Life Technologies).

### Bioinformatics for Ion Torrent

After generation, sequencing reads were filtered for quality and binned according to Ion Xpress barcode using Ion Torrent Suite software version 5.10.0. Sequencing reads in FASTQ format were further processed using web-based Galaxy software^14^. First, raw FASTQ files were normalized using the FASTQ groomer tool function. Next, each barcoded read was trimmed to remove the primer sequence and subsequently filtered to the expected size of the 16 s gene target. After this level of processing, the sequence reads were concurrently compared to the SILVA 16 s database using bowtie 2 software^15,16^. This yielded a call to genera level as well as the number of times each sequence matched the database (hit-rate). Where multiple calls to the same genera were made the number of hits were added accordingly. These numbers were then converted to percentage of total to give an overall ratio of the sequenced BES sample.

### Statistical analysis

Bacterial abundance (normalized per levels of 16S universal) was modeled by mixed analysis of variance with relation to the location and bacterium. Abundance levels were then normalized by dividing the raw abundance by the corresponding 16S universal abundance. This result was log (base-2) transformed to an improved approximation of normality prior to analysis. Differences among locations by bacterium were assessed by Tukey-adjusted contrasts. The bacterial levels of the uvula and endoscope were compared to each point along the esophagus and a pooled level of all points of the esophagus, including the Barrett’s tissue.

Relative proportions of bacteria at each location were calculated by determining the relative proportions of each bacterium in each patient, and then averaging the relative proportions of all patients afterward. Statistical analyses were performed using R statistical software (R Core Team, 2018, version 3.5.1). In all statistical tests, α = 0.05.

## Results

### Microbial composition

**Figure 2:**
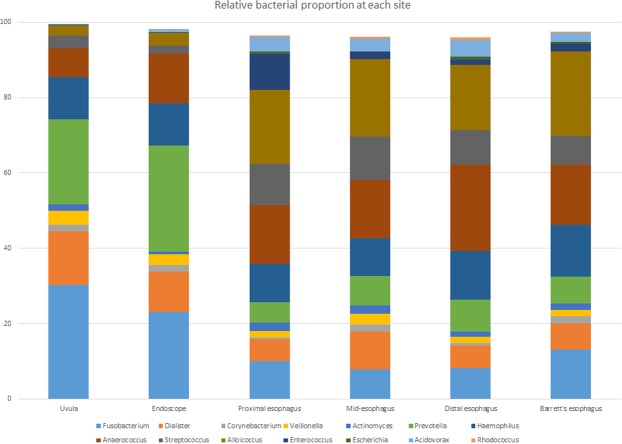
Relative abundance of microflora in uvula, endoscope and esophageal biopsies.

**Figure 3:**
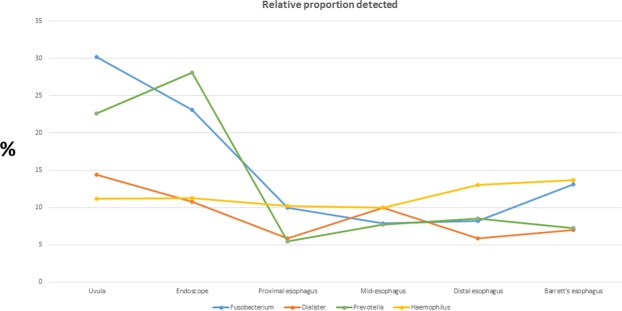
Relative proportion of *Fusobacterium*, *Dialister*, *Prevotella* and *Haemophilus* detected at uvula, endoscope and along esophagus.

**Figure 4:**
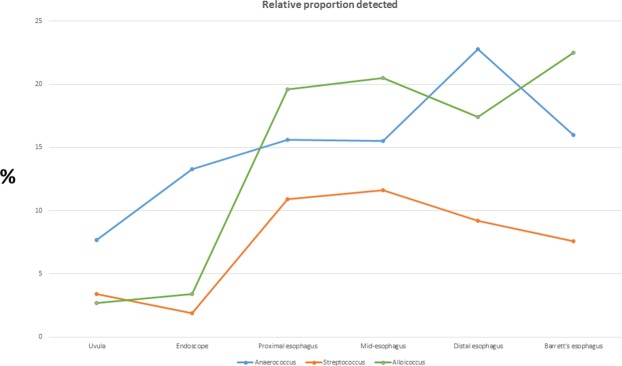
Relative proportion of *Anaerococcus*, *Streptococcus* and *Alloicocccus* detected at uvula, endoscope and along esophagus.

The relative proportions of microflora of the swabs and mucosal biopsies are shown in Fig. 2. There were wide differences in the microflora composition when comparing the uvula to each point of the esophagus and when comparing the endoscope swab to each point of the esophagus. *Fusobacterium*, *Prevotella*, and *Dialister* had the highest relative proportions in the uvula and on the endoscope, but were found in decreased proportions in the esophagus (Fig. 3). Anaerococcus, Streptococcus and Alloicoccus had the highest relative proportions in the esophagus, but were detected in lesser quantities in the uvula and on the endoscope (Fig. 4). There were only subtle differences in the microflora composition of the Barrett’s esophagus samples compared to the distal esophagus.

### Swabs vs esophageal biopsies

**Figure 5:**
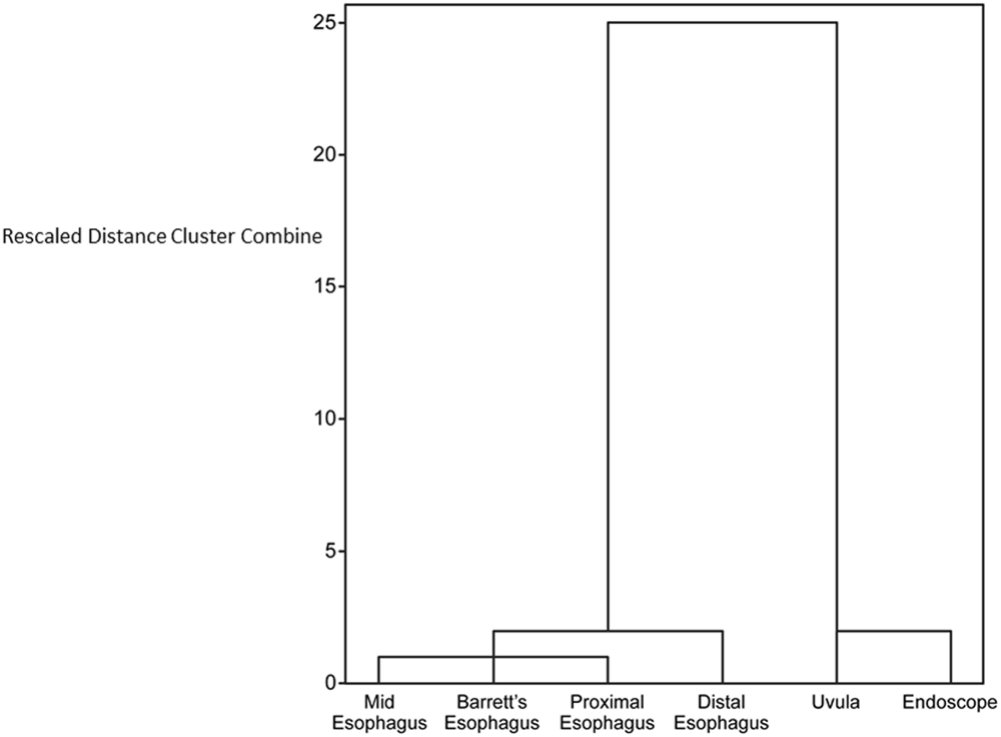
Cluster analysis of bacterial levels at uvula, endoscope and esophageal biopsies.

A hierarchical cluster analysis of the bacterial levels detected is shown in Fig. 5. The samples from the uvula and endoscope swabs clustered closely together, and were both relatively distinct from all of the mucosal biopsies along the esophagus.

### Uvula/endoscope vs. esophagus

**Figure 6:**
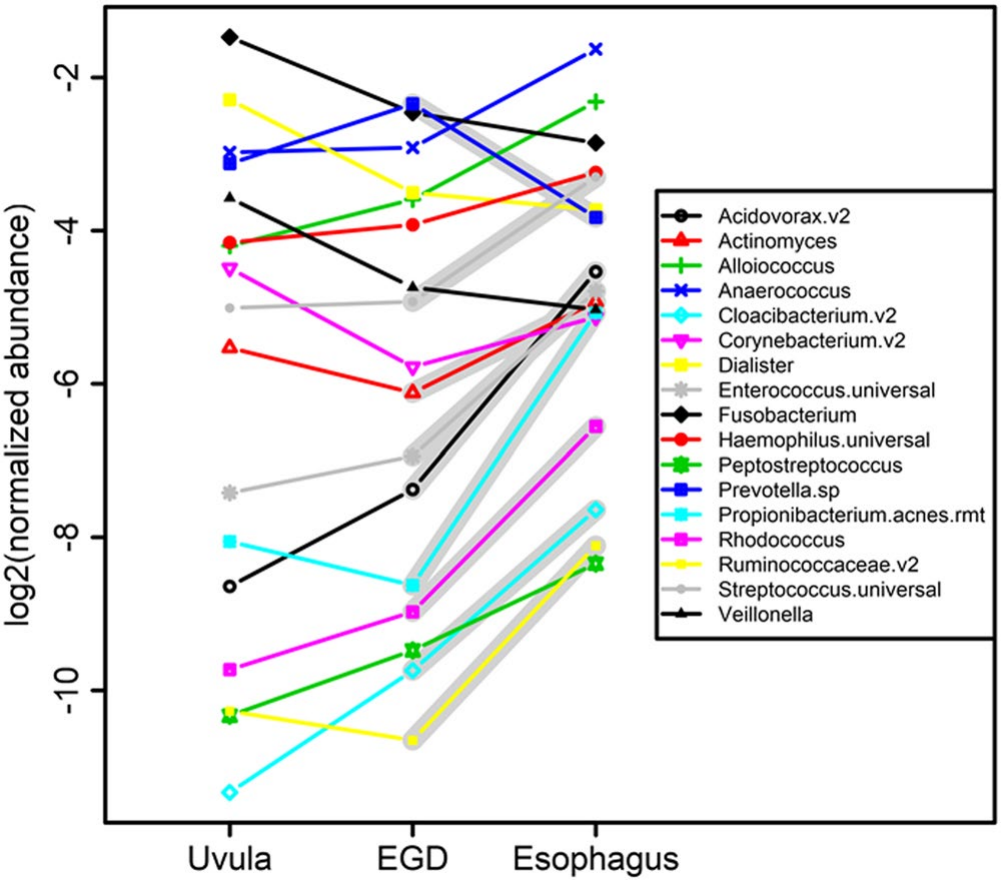
Normalized abundances of each bacterium in the uvula swab, endoscope swab or a pooled level of all esophageal points. A broad gray background indicates the bacteria which had statistically significant differences (p < 0.05) in levels between the uvula/endoscope and pooled esophagus.

We next examined the abundance levels of all detected bacteria. Figure 6 shows the normalized levels of all 17 identified bacteria. There were no statistically significant differences in any of these bacteria when comparing the uvula swab to the endoscope swab. There were 9 bacteria, however, which had statistically different levels in the esophagus compared to either the uvula or endoscope swab.

## Discussion

We undertook this simple but useful study to provide an evidence-based investigation of the best method to obtain esophageal specimens for microbiome analysis. Prior studies have demonstrated that the oral flora has a distinct microflora composition compared to the rest of the gastrointestinal tract. And although it has been previously shown that inflammation and metaplastic changes of the distal esophagus can be associated with alterations in the microbiome compared to the rest of the esophagus^17^, most studies have failed to examine the best methods to sample the microbiome along different aspects of the esophagus. As such, there have been numerous methods used to obtain samples to study the microbiome of the gastrointestinal tract. Some studies have used fecal samples to analyze the microbiome in diabetic patients^18^. Other studies have argued that the differences in the microbiome in the proximal and distal gastrointestinal tract are minimal compared to the differences between patients^8^. Multiple other techniques have been attempted to sample the gastrointestinal tract, ranging from fluid sampling to intraluminal brushing^19,20^. Given this lack of consensus and the wide variety of sampling techniques/locations used in previous literature, our goal was to determine how well the uvula and endoscope mimicked the proximal, mid and distal esophagus.

We decided to perform this study in patients with Barrett’s esophagus for several reasons. Firstly, as Barrett’s esophagus is a risk factor for esophageal cancer, it is likely that future analyses of the association of the microbiome and esophageal disease will utilize at least some patients with Barrett’s esophagus. Secondly, the patients with Barrett’s esophagus were going to have endoscopy with mucosal biopsy anyway for surveillance of their disease, so the additive risk from this study was extremely low. Thirdly, the amount of literature on the microbiome expression patterns in patients with Barrett’s esophagus is limited and we felt that there would be utility and interest in further elucidating the microflora in this patient population.

To date this type of study, investigating the similarity of uvula and endoscope swabs to the intraluminal environment along the esophagus, has not been performed. We felt that this study was important to determine if we could demonstrate that this type of microflora analysis could be performed while minimizing the risk of mucosal biopsy to the patient. From a clinical perspective, mucosal biopsy along the esophagus can occasionally be associated with bleeding and very rarely with perforation of the esophagus^21,22^. Furthermore, patients with Barrett’s esophagus have historically required up to 20 different biopsies in one setting according to the Seattle protocol^23^. The cumulative risk of this number of biopsies is not trivial. As such, many endoscopists may be hesitant to place a patient at increased risk by taking even more biopsies for research purposes. Our goal was to see if a swab of a part of the oropharynx, or the endoscope itself, could eliminate the need for a mucosal biopsy. Although we hypothesized that the uvula swab would not match the relatively acidic environment of the distal esophagus, we were unsure how similar it would be to the proximal esophagus. And we did feel that the endoscope itself may potentially have a similar microflora makeup compared to part or all of the esophagus. And we expected that the absolute abundance of the microflora could differ simply based on whether the sample was a swab or a mucosal biopsy. For this reason we included the relative abundances of each organism as well as the absolute abundances.

Previous studies analyzing the microbiome using only swabs or esophageal washes were unable to obtain a large enough concentration of bacteria for analysis^24^. Our study did show, however, that a simple swab of the uvula or even the endoscope itself would still have high enough levels of bacteria to perform analyses. One potential difference in our study was that we harvested the RNA immediately after collection, vs. freezing the specimen and then later performing the harvest. We recommend immediate harvest to obtain the highest yield of microflora.

Our study revealed that there is significant heterogeneity of the microflora at different aspects of the esophagus. It also showed that both the uvula and endoscope have very dissimilar relative microflora compositions compared to all aspects of the esophagus. This information is useful as investigators continue to explore the microbiome in the gastrointestinal tract. There is certainly an increased risk, albeit minimal, with taking additional biopsies for research purposes in a patient who may already be undergoing 16 to 20 biopsies. But it appears that the uvula and endoscope swabs are not able to match the mucosal biopsies, likely due to the vast differences seen in different parts of the esophagus. Changes in pH, motility, intraluminal pressure and other factors are likely to account for these differences.

We were interested in the microflora found within the Barrett’s esophagus compared to the distal esophagus. The differences were only very subtle. This similarity is perhaps expected, as the Barrett’s esophagus is directly adjacent to normal distal esophageal mucosa. It is likely that microflora composition, along with other intraluminal factors such as acidity, account for a particular focus of Barrett’s esophagus within the lumen.

We chose to focus on patients with Barrett’s esophagus because these patients were scheduled to receive a surveillance endoscopy for clinical purposes anyway. We wanted to minimize the risk to patients and not subject a patient to an endoscopy solely for research purposes. Though there may be some bias associated with all patients having Barrett’s esophagus, we feel that our primary research question was not significantly affected by using this cohort. Future studies will be enhanced, however, by adding patients who do not have Barrett’s esophagus. Including these patients into our study would help to see whether there were any particularly striking differences in patients with and without Barrett’s esophagus.

Our study had some limitations. Overall it was a small group of patients, but we were able to show significant differences both in the levels and relative proportion of the most prevalent organisms detected. We also had a relatively limited panel consisting of 34 bacteria. But many of the most prevalent organisms identified in previous literature, such as *Streptococcus*, *Prevotella*, *Fusobacterium and Veillonella*, were on the panel. Although we feel that the panel we used was appropriate for our research question, future studies will likely benefit from use of a broader panel of organisms.

In conclusion, mucosal biopsies should remain the gold standard for microflora analysis in the esophagus. Though less invasive, uvula and esophageal swabs do not provide a good replacement for the mucosal biopsies. And there is heterogeneity in the microflora composition in various aspects of the esophagus. In the future, there will be ever-increasing investigation into the role of microflora in esophageal disease. Obtaining an accurate representation of the bacterial composition will be critical in advancing our knowledge base in this field.

## Data Availability

The dataset generated from this study is available upon request.
